# Mitochondria-targeted hydrogen sulfide attenuates endothelial senescence by selective induction of splicing factors *HNRNPD* and *SRSF2*

**DOI:** 10.18632/aging.101500

**Published:** 2018-07-19

**Authors:** Eva Latorre, Roberta Torregrossa, Mark E. Wood, Matthew Whiteman, Lorna W. Harries

**Affiliations:** 1 University of Exeter Medical School, University of Exeter, UK; 2 College of Life and Environmental Sciences, University of Exeter, UK

**Keywords:** splicing factors, senescence, H_2_S, AP39, AP123, RT01, mitochondria-targeting, persulfide, perthiol

## Abstract

Cellular senescence is a key driver of ageing, influenced by age-related changes to the regulation of alternative splicing. Hydrogen sulfide (H_2_S) has similarly been described to influence senescence, but the pathways by which it accomplishes this are unclear.

We assessed the effects of the slow release H_2_S donor Na-GYY4137 (100 µg/ml), and three novel mitochondria-targeted H_2_S donors AP39, AP123 and RT01 (10 ng/ml) on splicing factor expression, cell proliferation, apoptosis, DNA replication, DNA damage, telomere length and senescence-related secretory complex (SASP) expression in senescent primary human endothelial cells.

All H_2_S donors produced up to a 50% drop in senescent cell load assessed at the biochemical and molecular level. Some changes were noted in the composition of senescence-related secretory complex (SASP); IL8 levels increased by 24% but proliferation was not re-established in the culture as a whole. Telomere length, apoptotic index and the extent of DNA damage were unaffected. Differential effects on splicing factor expression were observed depending on the intracellular targeting of the H_2_S donors. Na-GYY4137 produced a general 1.9 – 3.2-fold upregulation of splicing factor expression, whereas the mitochondria-targeted donors produced a specific 2.5 and 3.1-fold upregulation of *SRSF2* and *HNRNPD* splicing factors only. Knockdown of *SRSF2* or *HNRNPD* genes in treated cells rendered the cells non-responsive to H_2_S, and increased levels of senescence by up to 25% in untreated cells.

Our data suggest that *SRSF2* and *HNRNPD* may be implicated in endothelial cell senescence, and can be targeted by exogenous H_2_S. These molecules may have potential as moderators of splicing factor expression and senescence phenotypes.

## Introduction

Ageing is characterised by a progressive decline of physiological function accompanied by increased incidence of age-related disease. The biochemical and functional pathways most dysregulated by age in the human peripheral blood transcriptome are enriched for transcripts encoding the regulatory machinery that governs splice site choice [1]. Changes in splicing regulation have also been linked with lifespan in both mammalian and invertebrate model systems [2,3]. Evidence that these changes are functional is provided by the observation that large-scale dysregulation of patterns of alternative splicing is characteristic of many age related diseases such as Alzheimer’s disease [4], Parkinson’s disease [5] and cancer [6]. These observations highlight the importance of maintenance of correct splicing regulation for health throughout the life course [7].

The accumulation of senescent cells is emerging as an important driving factor of the ageing process in multiple species [8–11]. Senescent cells do not divide, are viable and metabolically active, but have altered physiology. This includes the secretion of the SASP, a cocktail of pro-inflammatory cytokines and tissue remodelling factors that induces senescence in neighbouring cells in a paracrine manner [12]. Senescent cells in endothelium and cardiac tissues have been associated with increased cardiovascular dysfunction [13]. Senescence of cardiomyocytes and endothelial cells has been associated with hardening of the heart muscle and stiffening of the vascular wall, resulting in angina, dyspnea and heart failure [14]. Endothelial cell senescence has also been associated with vascular dysfunction and increased vascular risk [15]. Perhaps most persuasively, targeted removal of senescent cells in transgenic mouse models has been shown to result in improvements to multiple ageing phenotypes [16,17]. Senescent cells also show dysregulation of splicing regulator expression in vitro [18,19], and restoration of splicing factor expression to levels comparable with those seen in younger cells has recently been demonstrated to be associated with reversal of multiple senescence phenotypes in senescent human primary fibroblasts [20].

There is now enormous interest in compounds with the potential to kill senescent cells (senolysis) or ameliorate their effects (senostasis). The endogenous gaseous mediator hydrogen sulfide (H_2_S) has been described to exert a protective effect against cellular senescence and ageing phenotypes [21–23], and accordingly, to have protective effects against several age related diseases [24–27], although many of these studies have been carried out using non-physiological conditions, using very high levels of H_2_S. Plasma H_2_S level declines with age [28], is associated with hypertension in animals and humans [21,29] and shows a significant inverse correlation with severity of coronary heart disease [30]. Disruption of H_2_S homeostasis may also contribute to the pathogenesis of atherosclerosis [31], where H_2_S could play an anti-atherogenic role [32]. Conversely, supplementation of animals with an exogenous source of H_2_S reverses the disease phenotype [33]. H_2_S has been proposed to prevent cell damage under stress in part due to persulfidation of target proteins [34]. These observations suggest that H_2_S could represent a potential new intervention for ageing and age-related disease.

Here, we aimed to assess the effect of the H_2_S donor Na-GYY4137 [35,36], and since mitochondria are a source and a target of H_2_S, three novel H_2_S donors, AP39, AP123 and RT01 previously demonstrated to be targeted specifically to the mitochondria [37–39], on splicing regulatory factor expression and cell senescence phenotypes in senescent primary human endothelial cells. Treatment with Na-GYY4137 resulted in an almost global upregulation of splicing factor expression in treated cells consistent with that observed with resveratrol analogues in our previous work [20]. Conversely, H_2_S donors targeted to the mitochondria also resulted in rescue from senescence but each demonstrated a very specific upregulation of transcripts encoding the splicing activator protein SRSF2 and the splicing inhibitor protein HNRNPD. Abolition of either *SRSF2* or *HNRNPD* expression in primary endothelial cells by morpholino technologies in the absence of any treatment resulted in increased levels of cellular senescence. None of the H_2_S donors were able to reduce senescent cell load in cells in which *SRSF2* or *HNRNPD* expression had been abrogated. These data strongly suggest that mitochondria-targeted H_2_S is capable of rescuing senescence phenotypes in endothelial cells through mechanisms that specifically involve *SRSF2* and *HNRNPD*.

## RESULTS

### Treatment with H2S donors partially reversed several senescence phenotypes

**Figure 1:**
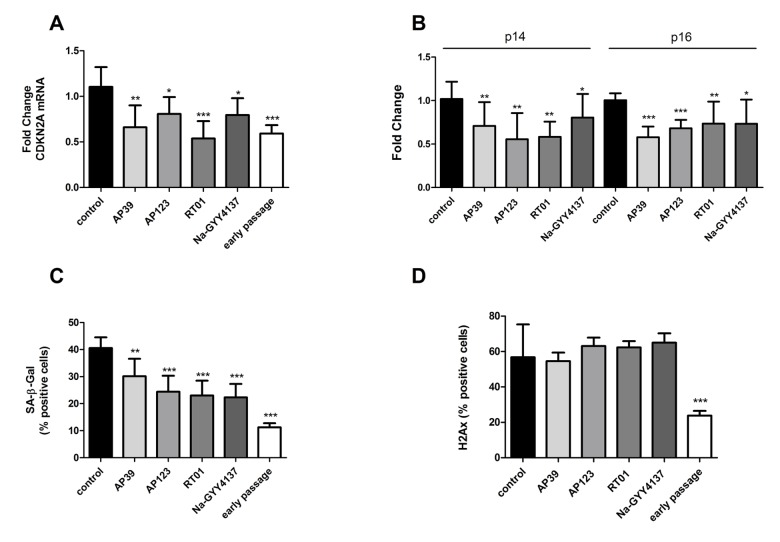
**H_2_S donor treatment is associated with partial rescue from cellular senescence phenotypes.** Levels of the senescence-associated total *CDKN2A* gene expression (**A**) and levels its alternatively-expressed isoforms p14 and p16 (**B**) were assessed by qRTPCR in senescent endothelial cells after 24h treatment with H_2_S donors (Na-GYY4137 at 100 µg/ml, AP39, AP123, RT01 at 10 ng/ml). Data are expressed relative to stable endogenous control genes *GUSB*, *IDH3B* and *PPIA*, and are given normalised to the levels of the individual transcripts as present in vehicle-only treated control cells. Fold change was calculated for in triplicate for three biological replicates. (**C**)**.** The proportion of cells staining positive for Senescence Associated β-galactosidase (SA-β-Gal) activity following treatment with H_2_S donors was determined by manually counting the percentage of SA-β gal positive cells. (**D**) The proportion of cells staining positive for H2Ax marker of DNA damage following treatment with H_2_S donors was determined by manually counting the percentage of H2Ax positive cells. N = >300 cells for each sample. Statistical significance is indicated by *p<0.05, ** p<0.005, *** p<0.0001 (2 way ANOVA).

**Figure 2:**
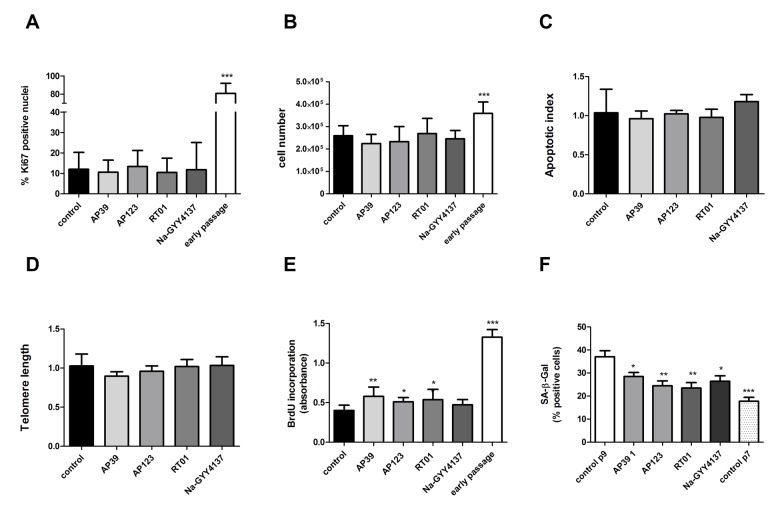
**Cell proliferation rate is not affected by H_2_S donor treatment.** (**A**) Proliferation index was assessed for treated cells as assessed by Ki67 immunofluorescence (>400 nuclei counted per sample). (**B**) Cell counts following 24h treatment with Na-GYY4137 at 100 µg/ml, AP39, AP123, RT01 at 10 ng/ml. (**C**) Apoptotic index in senescent cells treated with inhibitors as determined by TUNEL assay. Data are derived from duplicate testing of 3 biological replicates. (**D**) Telomere length was assessed by qPCR in three biological and 3 technical replicates. (**E**) BrdU incorporation into cellular DNA. Relative BrdU incorporation was assessed in 3 biological replicates and was calculated by normalization of data to values corresponding to untreated (control) cells and are expressed as % BrdU incorporation. (**F**) Effect of 24h treatment with Na-GYY4137 at 100 µg/ml, AP39, AP123, RT01 at 10 ng/ml on accumulation of senescent cells over 2 passages in early passage cells (PD = 44). Mean+- SD of three independent experiment is shown. Statistical significance is indicated by *** p<0.001. Error bars represent the standard error of the mean.

We treated senescent human primary endothelial cells with the H_2_S donor Na-GYY4137 at 100 µg/ml or with each of the mitochondrially-targeted H_2_S donors AP39, AP123 or RT01 (10ng/ml) for 24 hours and then assessed cellular senescence by molecular and biochemical means as described above. We used a 10,000 fold lower concentration of the mitochondrial compounds compared to Na-GYY4137, since our previous studies have indicated these molecules to be considerably more potent that non-targeted H_2_S [38,40]. We identified a decrease in total *CDKN2A* expression of up to 50% (Figure 1A) compared with vehicle-only control. The decrease in expression was comparable for both p16 and p14 isoforms of the *CDKN2A* gene (Figure 1B). These molecular changes were accompanied by a 25 to 40% decrease in the senescent cell fraction following treatment with any of the H_2_S donors tested (Figure 1C). We also determined that levels of DNA damage were unaffected in H_2_S donor- treated cells (Figure 1D). To assess whether the reduction in senescent cell load was due to an increase in the proliferative capacity of the cells or a selective killing of senescent cells, we examined rates of proliferation and apoptosis. We identified no increase in Ki67 staining (indicative of cell proliferation [41]; or in cell number, indicating that the cultures as a whole had not regained proliferative capacity (Figures 2A and 2B). We did note a very small but significant increase in levels of S-phase cells by BrdU staining, indicating that a small percentage of the culture had recommenced DNA replication (Figure 2C). No increase in levels of apoptosis was observed in the treated cell cultures (Figure 2D), indicating that the reduction in senescent cell load was not due to a selective killing of senescent cells. No restoration of telomere length was evident in H_2_S donor-treated cells (Figure 2D). Initial evidence also suggests that treatment with H_2_S donors may be able to bring about retardation of senescence as well as reversal. Early passage cells seeded at PD = 44 treated with H_2_S donors demonstrated a reduction in the number of SA-β-Gal positive cells two passages later (Figure 2F).

### Treatment with H_2_S donors caused alterations to splicing factor expression

**Figure 3:**
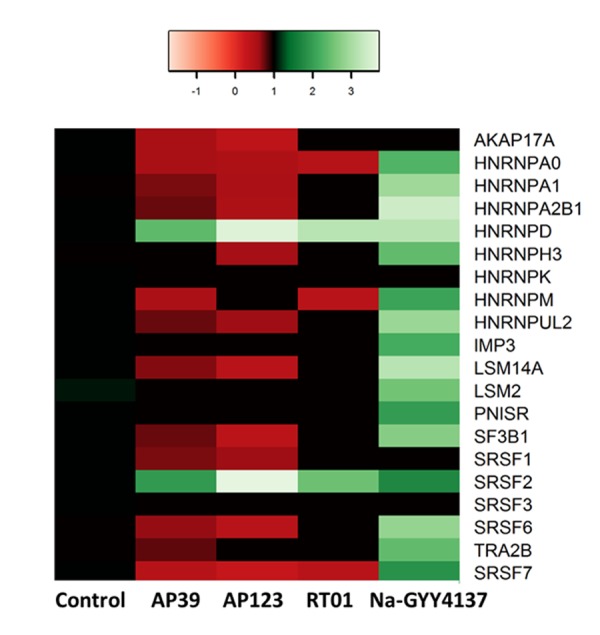
**H_2_S donor treatments affect splicing factor transcript expression.** The change in splicing factor mRNA levels in response to 24hr treatment with H_2_S donors are given ; Na-GYY4137 at 100 µg/ml, AP39, AP123, RT01 at 10 ng/ml. Green indicates up-regulated genes, red denotes down-regulated genes. The colour scale refers to fold-change in expression. Only statistically significant changes are presented in the heat map.

To establish whether H_2_S donors could influence splicing regulators, we first measured splicing factor expression by qRT-PCR in senescent cultures of endothelial cells following 24hr treatment with AP39, AP123, RT01 (at 10ng/ml) and Na-GYY4137 at 100 µg/ml. We determined that treatment with Na-GYY4137 led to an increase in splicing factor expression comparable with that we had previously observed following treatment with the stilbene molecule resveratrol or its analogues [20] (Figure 3 and Table 1). Levels of splicing factor expression were also consistent with levels we had previously observed in untreated young cells [18]. This was a generalised effect on splicing factors, with the majority showing altered expression as in our previous work with resveratrol analogues [20]. Conversely, treatment with all of the mitochondria-targeted donors led to a very specific increase in the expression of *SRSF2* and *HNRNPD* genes, whereas the majority of the other splicing factors demonstrated reduced expression (Figure 3).

**Table 1:** The effects of treatment with H_2_S donors on splicing factor expression.

	**Control**	**AP39**	**AP123**	**RT01**	**GYY4137**
***AKAP17A***	1.007 (0.069)	0.572* (0.099)	0.367** (0.045)	0.582 (0.216)	2.016 (0.920)
***HNRNPA0***	1.006 (0.064)	0.560** (0.048)	0.521** (0.046)	0.434** (0.081)	2.269*** (0.120)
***HNRNPA1***	1.000 (0.021)	0.717*** (0.033)	0.539*** (0.001)	0.667 (0.226)	2.909* (0.593)
***HNRNPA2B1***	1.002 (0.039)	0.766* (0.084)	0.532** (0.016)	0.823 (0.249)	3.391* (1.006)
***HNRNPD***	1.021 (0.026)	2.374* (0.221)	3.618** (0.052)	3.171* (0.681)	3.188* (0.548)
***HNRNPH3***	1.000 (0.018)	0.867 (0.166)	0.599** (0.111)	0.627 (0.192)	2.404* (0.464)
***HNRNPK***	1.012 (0.089)	1.193 (0.106)	0.804 (0.115)	1.324 (0.257)	1.207 (0.149)
***HNRNPM***	1.018 (0.109)	0.552* (0.064)	0.641 (0.160)	0.423* (0.135)	2.061* (0.380)
***HNRNPUL2***	1.002 (0.036)	0.751*** (0.018)	0.621** (0.100)	0.871 (0.301)	2.862* (0.631)
***IMP3***	1.004 (0.051)	0.980 (0.094)	1.111 (0.155)	0.889 (0.099)	2.182** (0.270)
***LSM14A***	1.005 (0.057)	0.696** (0.058)	0.428** (0.012)	0.602 (0.188)	3.156* (0.635)
***LSM2***	1.079 (0.235)	0.974 (0.074)	1.009 (0.192)	1.346 (0.365)	2.526* (0.409)
***PNISR***	1.008 (0.075)	0.951 (0.078)	0.707 (0.093)	0.672 (0.228)	1.991* (0.388)
***SF3B1***	1.003 (0.039)	0.758** (0.029)	0.406*** (0.000)	0.692 (0.283)	2.695* (0.690)
***SRSF1***	1.004 (0.054)	0.707** (0.039)	0.640* (0.195)	0.632 (0.194)	1.404 (0.290)
***SRSF2***	1.002 (0.039)	1.967* (0.332)	3.704*** (0.169)	2.496** (0.312)	1.724** (0.117)
***SRSF3***	1.013 (0.093)	1.231 (0.139)	1.362 (0.307)	0.888 (0.189)	1.368 (0.149)
***SRSF6***	1.000 (0.005)	0.655* (0.110)	0.413*** (0.013)	0.524 (0.199)	2.831* (0.786)
***TRA2B***	1.000 (0.010)	0.773** (0.045)	1.010 (0.329)	0.650 (0.233)	2.396** (0.383)
***SRSF7***	1.005 (0.059)	0.441*** (0.356)	0.286** (0.010)	0.424* (0.157)	1.862* (0.543)

Changes to mRNA levels in senescent primary human endothelial cells in response to treatment with H_2_S donors (Na-GYY4137 at 100ug/ml) or AP39, AP123, RT01 at 10ng/ml) for 24h. Data are derived from duplicate testing of 3 biological replicates. In red is indicated that common effect of the H_2_S donors. Standard deviation (SD) is given in parentheses. Statistically significant results are indicated in bold typeface, and significance level is indicated by stars *p<0.05, **p<0.005 and ***p<0.0001 compared with the corresponding control value.

### Treatment with H_2_S donors caused a consistent increase in the release of IL8

We then set out to determine if treatment with H_2_S donors had an impact on the SASP in senescent cultures of endothelial cells treated for 24 hours with AP39, AP123, RT01 (at 10 ng/ml) and Na-GYY4137 at 100 µg/ml. Levels of multiple cytokines including key SASP components (IL6, IL8, TNFα, IL2, IL1β, IL12p70, IL10, INFγ and GMCSF) were determined in senescent endothelial cell supernatants by ELISA. We noted altered expression of GM-CSF, IL12, IL2, IL6 and IL8 in senescent endothelial cells, which is comparable with our previous work in this cell type [19]. Upon treatment with H_2_S donors, the only consistent change for all donors was an upregulation of IL8 expression, although this was not to levels comparable with younger passage cells (Table 2). The other cytokines were unaffected by treatment with any of the donors.

**Table 2:** The Effect of H2S donors on expression of Senescence-Associated Secretory Phenotype (SASP) proteins.

	**Control**	**Young Cells**	**AP39**	**AP123**	**RT01**	**GYY**
**GM-CSF**	2.915 (0.241)	3.444 (0.243)	3.008 (0.391)	3.659 (0.213)	3.651 (0.159)	10.410 (0.455)***
**INFg**	41.099 (3.267)	41.439 (2.984)	38.346 (3.727)	29.774 (5.240)	45.805 (7.065)	40.817 (3.337)
**IL10**	0.299 (0.059)	0.412 (0.061)	0.441 (0.069)	0.387 (0.061)	0.477 (0.067)	0.329 (0.034)
**IL12p70**	0.797 (0.173)	0.832 (0.169)	0.561 (0.145)	0.959 (0.314)	0.811 (0.245)	0.515 (0.052)**
**IL1b**	0.317 (0.182)	0.176 (0.039)	0.172 (0.047)	0.283 (0.054)	0.217 (0.074)	0.280 (0.044)
**IL2**	0.713 (0.268)	1.008 (0.583)	1.130 (0.287)	0.449 (0.167)	0.980 (0.230)	1.487 (0.186)***
**IL6**	1431.728 (11.463)	1433.852 (12.712)	1439.876 (46.458)	1454.688 (41.138)	1454.689 (41.138)	710 (35.799)***
**IL8**	5885.113 (349.38)	6750.428 (156.941)***	7342.547 (295.224)***	7461.439 (210.010)***	7559.555 (327.008)***	17321.310 (631.756)***
**TNFa**	4.592 (0.512)	5.463 (0.713)	4.974 (0.582)	4.489 (1.124)	5.211 (0.602)	4.443 (0.059)

Protein levels of components of the senescence associate secretory phenotype (SASP) were determined using Mesoscale ELISA platform in the culture medium from senescent endothelial cells treated with Na-GYY4137 (at 100ug/ml) or AP39, AP123, RT01 (at 10ng/ml) for 24 hours and from an early passage (Young cells) PD24. The results are expressed as pg/ml of 3 independent experiments. Control refers to vehicle-only treated cells. Standard Deviation (SD) is given in parentheses. Statistically-significant findings are indicated in bold typeface, and significance level is indicated by stars with **p<0.005 and ***p<0.001 compared with the corresponding control value (senescent endothelial cells).

### HNRNPD and SRSF2 are necessary for H_2_S donor-associated reduction in senescent cell load

**Figure 4:**
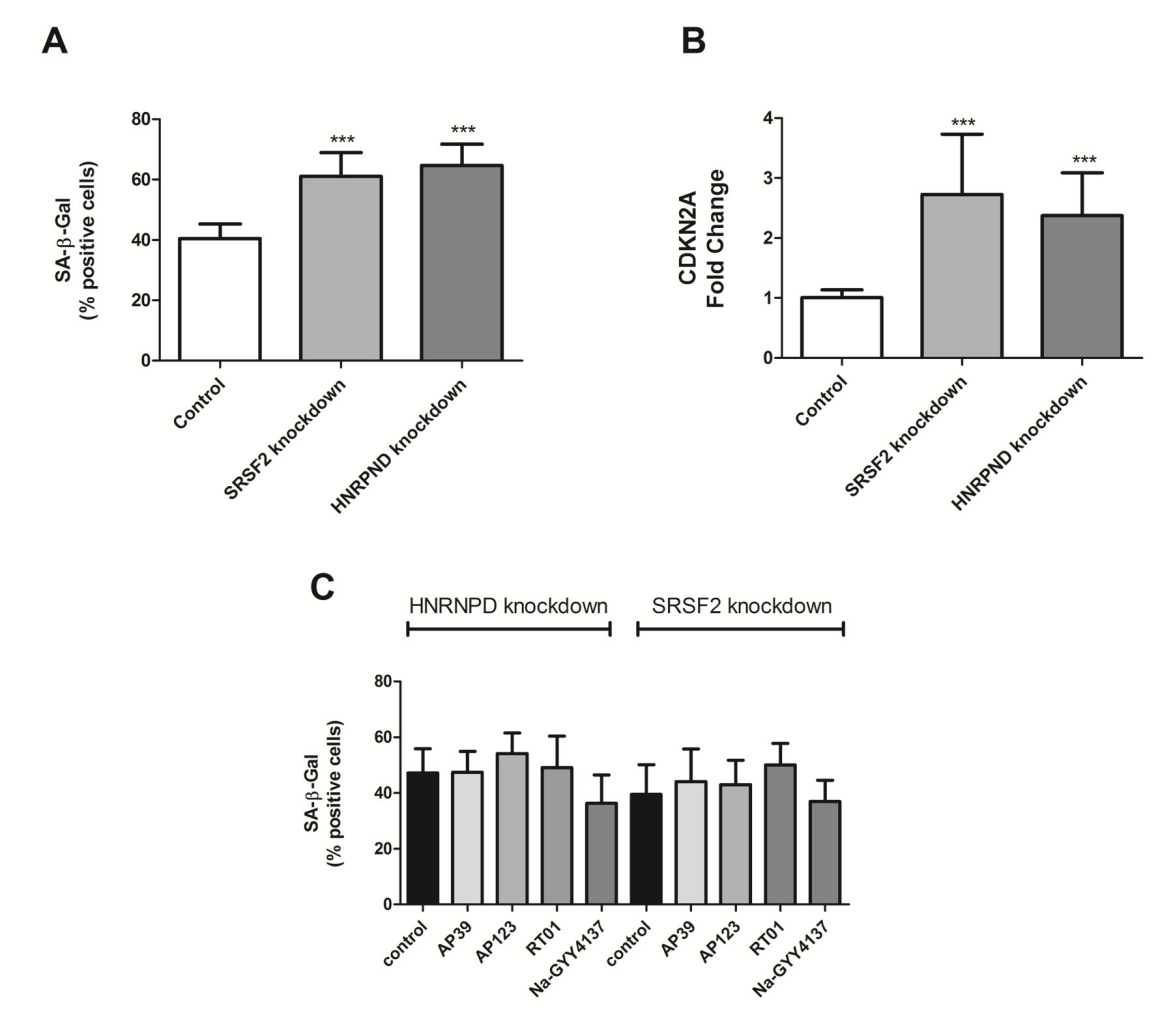
**The cellular and molecular effects of targeted knockdown of HNRPND and SRSF2 genes.** (**A**) Senescent cell load as indicated by SA-β-Gal staining following either *HNRPND* or *SRSF2* gene knockdown. n>300 cells for each sample. (**B**) Senescent cell load as indicated by *CDKN2A* gene expression following *HNRPND* or *SRSF2* gene knockdown. Data are expressed relative to stable endogenous control genes *GUSB, IDH3B* and *PPIA*, and normalised to the levels of the individual transcripts in vehicle only controls. (**C**) The effect of *HNRPND* or *SRSF2* gene knockdown on senescent cell load after H_2_S donor treatment. Data are derived from duplicate testing of 3 biological replicates. Statistical significance is indicated by *** p<0.001. Error bars represent the standard error of the mean.

A significant increase of *HNRNPD* and *SRSF2* were the only consistent changes in splicing factor expression produced by treatment with any H_2_S donor tested. We did not test every known splicing factor for effect, electing to assay only those where we had á priori evidence for a role in senescence from our previous work [1,2,18–20]. We therefore aimed to test the role of *SRSF2* and *HNRNPD* in determination of senescent cell phenotypes using gene knockdown technologies. Knockdown for *SRSF2* and *HNRNPD* genes was 60% and 52% respectively as measured by immunofluorescence. We first assessed the effect of knockdown of *SRSF2* or *HNRNPD* expression in primary human endothelial cells on SA-β-Gal staining and total *CDKN2A* expression. Knockdown of either *SRSF2* or *HNRNPD* expression was sufficient to cause an increase in the numbers of senescent cells from 40% to 60% and 63% for *HNRNPD* and *SRSF2* respectively (Figure 4A). Dual knockdown of both genes resulted in cell death. Similar effects were noted for *CDKN2A* expression, a molecular marker of senescence (Figure 4B). Finally, following knockdown of either *SRSF2* or *HNRNPD*, cells were now unable to respond to any of the H_2_S donors (Figure 4C), which strongly suggests that the partial reversal of senescence phenotypes we have noted involves, at least in part, the action of these two genes.

## DISCUSSION

The accumulation of senescent cells is a key driver of ageing phenotypes. Accordingly, much interest is now focused on interventions that are able to selectively remove senescent cells (senolysis) or to attenuate their phenotype (senostasis). In the work described here, we demonstrate that treatment compounds which release low levels of the endogenous cytoprotective gas H_2_S was able to attenuate the senescent cell phenotype in late passage primary human endothelial cells, consistent with previous reports in senescent fibroblasts [42] where bolus sulfide was used. In our study we have used novel slow release compounds and target the key organelles regulating cell fate, the mitochondria. We observed a reduction in levels of senescent cells, as evidenced by a reduction in levels of the biochemical senescence marker SA-β-Gal, with concordant decreases in level of *CDKN2A* transcripts, a molecular marker of senescence. Initial experiments using treatment of early passage non-senescent cells suggests that treatment may be able to retard, as well as partially reverse senescence. Although this is not an exhaustive experiment, and requires longer term assessment to definitively reach a conclusion, it suggests that the rate of accrual of senescent cells in treated cultures may be slower. Some changes to the composition of the SASP were also apparent, with the predominant finding being elevation of IL-8 expression, but no effects on cell proliferation were noted and splicing factors were not investigated. These changes were accompanied by changes in the profile of transcripts encoding components of the splicing regulatory machinery, which showed differential effects depending on whether the H_2_S donors were targeted to the mitochondria or not targeted for specific organelles. Rescue of senescence by novel mitochondria-targeted H_2_S donors was specifically mediated through changes in the expression of *SRSF2* and *HNRNPD* splicing factors, implicating a new interplay between mitochondrial biology, H_2_S, senescence and regulation of the splicing repertoire.

In contrast to our earlier work [20], the partial rescue of senescence brought about by treatment with H_2_S donors was not accompanied by major re-entry to cell cycle; this may reflect ongoing growth arrest through paracrine signalling, since many components of SASP were unaltered. A lack of renewed proliferation is not a negative outcome, since these cells are still aged and may have a significant mutational load. A very small percentage of the cells in the culture may recommence S-phase, as indicated by the small but significant increase in BrdU staining in the treated cultures. This may represent a subset of senescent cells that are capable of recommencing cell cycle, but the contribution of these cells to the senescent cell population is very minor. These data do however indicate a degree of uncoupling between senescence rescue and the ability to proliferate, which has been previously demonstrated for p21 deficient mouse fibroblasts. In this case, p21^-/-^ cells displayed most other hallmarks of senescence, yet failed to undergo cell cycle arrest at the G1 checkpoint [43].

Similarly, although the attenuation of SASP response was not sufficient lead to large scale cell cycle re-entry, it may be sufficient to induce differences in the functional characteristics of treated cells. Endothelial cells are a specialised cell type, characterised by structural and functional heterogeneity, and the shape and organization of cells vary across the vascular tree [44]. SASP differs from cell type to cell type; and the SASP of endothelial cells differs from that of other cell types such as fibroblasts and cardiomyocytes [19,20]. Treated endothelial cells express increased levels of IL8 compared to untreated cells, but no modifications to levels of other cytokines [19]. IL8 is an important pro-inflammatory cytokine, but also is known to be a potent promoter of angiogenesis. However, elevated IL-8 expression alone is not enough for induction of proliferation [45]. Moreover, IL-8 is a direct upstream regulator of vascular endothelial growth factor (VEGF) which plays a central role in angiogenesis and vascular maintenance [46]. From this we conclude that H_2_S has a senostatic, rather than a senolytic or a proliferation-inducing function in the majority of senescent cells in the culture.

Splicing factors are also known to influence the ageing process; the pathways most altered in the human peripheral blood transcriptome are enriched for genes encoding splicing factors and other regulators of gene expression [1] and dysregulation of splicing factor expression is also a characteristic of senescent cells [18]. Restoration of splicing factor expression in senescent human fibroblasts to levels consistent with those found in younger cells is sufficient to bring about cellular rejuvenation [20]. Senescence-related splicing changes have also been associated with the prevalence of coronary heart disease [19]. We hypothesise that a reduction in the fine-tuning of gene expression may contribute to attenuated stress responses in aged human cells leading ultimately to the development of age-related disease [7]. Our data reveal differential effects of H_2_S donors in senescent endothelial cells when targeted to the mitochondria compared with Na-GYY4137, which has no targeting. Treatment with Na-GYY4137 resulted in partial rescue of senescence and upregulation of levels of most splicing factors tested (Figure 3), as we have previously observed for other small molecule moderators of splicing factor expression [20]. Conversely, treatment with mitochondria-targeted donors, used at 10,000 fold lower concentration, was associated with a very specific upregulation of *SRSF2* and *HNRNPD*. *SRSF2* is a key splicing activator in endothelial cells [47], whereas *HNRNPD* may play an important role in senescence by virtue of its known role in the regulation of telomerase [48]. Rescue from senescence upon H_2_S donor treatment was abolished when expression either *SRSF2* or *HNRNPD* expression was reduced using morpholino technologies (Figure 4), indicating that these two genes may play a pivotal role in the senostatic response of cells to H_2_S. Furthermore, these genes may be the predominant link between splicing regulation and senescence in this cell type, since knockdown of either gene in untreated cells is associated with elevated levels of senescence.

The mechanistic basis for our observations is less clear cut. H_2_S has a known effect on antioxidant status [49,50]. AP39 and AP123 have previously been shown to reverse oxidative stress-induced mitochondrial oxidant production and normalise cellular bioenergetics in endothelial cells (37, 38), kidney epithelial cells [51] and cortical neurons [52] *in vitro* and *ex vivo* as well as myocardial cells [53] and cortical neurons [54] *in vivo*. Direct ‘oxidant scavenging’ and antioxidant mechanisms have been suggested to be key processes in preventing and/or reversing senescence and the literature is replete with the beneficial effects of antioxidants in this context (for example [55–60]. However, it is unlikely that direct ‘oxidant scavenging’ or antioxidant effects of by H_2_S were responsible for the protective observations in our study since the rate constants for reaction of H_2_S even as a bolus of up to 5 mM sulfide (NaSH; i.e. not generated slowly at very low levels as in our current study) with biologically relevant oxidant species are too low for direct antioxidant / radical scavenging effects [61] e.g. *vs* intracellular oxidant species peroxynitrite (*k* = 4.8 x 10^3^ M^-1^ s^-1^), hydrogen peroxide (*k* = 0.73 M^-1^ s^-1^), superoxide (*k* = 200 M^-1^ s^-1^) [61,62] etc. It is possible ‘scavenging’ of hydroxyl radicals could have occurred (*k* = 1.5 x 10^-7^ M^-1^ s^-1^ [61];) but considering the very low cellular concentrations of endogenous H_2_S or H_2_S generated very slowly from our donor molecules (37, 38) and used in our current study at very low concentrations (e.g. 10 ng/ml), this is unlikely a major mechanism for cytoprotection and direct reactions with oxidants probably do not account for the protective effects of slowly generated H_2_S in our system using our donor molecules [61].

H_2_S has also been suggested to exert some of its beneficial effects through SIRT1 [23,63], where treatment with H_2_S donors has been demonstrated to abolish oxidative stress in cardiomyocytes via SIRT upregulation [64]. H_2_S has also been reported to attenuate inflammation partially by promoting SIRT3 [49]. However, this study used a bolus of commercial NaSH at high (physiologically unattainable) concentrations, rather than the use of enzymatically-generated H_2_S such as we have used here, so these results must be interpreted with caution. SIRT1 activation is also unlikely to explain the changes in splicing factor expression we have observed, since SIRT1-null resveralogues still moderated splicing factor changes in human senescent fibroblasts [20], and effects of splicing modifier compounds are still evident in SIRT null cells [65].

One potential answer may lie in the ability of H_2_S donors to modify thiol groups of specific cysteines in target proteins *via S*-persulfidaton (‘sulfhydration’). *S*-Persulfidation has been shown to contribute to the changes of DNA methylation, DNA damage and repair and transcription factors, among others [66,67]. H_2_S is known to S-persulfidate the signalling protein MEK, which confers protection from cell senescence [68] and mitochondria-targeted H_2_S donors such as AP39 specifically persfulfidate mitochondrial proteins [69]. We have previously implicated the ERK kinase, just downstream of MEK in RAS/REF/MEK/ERK signalling, in control of splicing factor expression and senescence rescue [20]. It is possible therefore that H_2_S may attenuate ERK activity through S-persulfidation of its regulator, MEK. H_2_S, at albeit high concentrations generated from NaSH (a non-targeted source of H_2_S), is also able to *S*-persulfidate ATP synthase which resulted in increased catalytic activity (i.e. ATP synthesis) [70], previously implicated in lifespan extension in *Drosophila* species by interaction with both mTOR and MEK/ERK pathways [71]. An interaction between MEK/ERK and ATP synthase may also explain the differential effects of mitochondrial-targeted and non-targeted donors on splicing factor expression. H_2_S targeted directly to the mitochondria would be expected to produce more localised dosing and larger effects; signalling pathways commonly exhibit dose and context effects [72].

A caveat of our study is that senescent cell populations in culture are heterogeneous, consisting of senescent cells of different subtypes, but also non-senescent cells which have become growth arrested because of the effects of paracrine signalling by virtue of SASP. It is very difficult to achieve a 100% senescent culture, so our measurements represent population averages, not dynamics of individual senescent cells. It should also be noted that although *SRSF2* and *HNRNPD* demonstrate a specific response to H_2_S targeted to the mitochondria, most splicing factors probably would have capacity to produce the effects we have seen if they had been responsive, as we have demonstrated in our previous work [20]. It should also be noted that we cannot rule out the involvement of other splicing factors that we have not tested for H_2_S response.

We conclude from these data that treatment with H_2_S is able to rescue some features of cellular senescence in late passage human primary endothelial cells, and that this phenomena (in the case of the mitochondria-targeted donors AP39, AP123 and RT01) may be mediated through the action of the *SRSF2* and *HNRNPD* splicing factors. These observations suggest that modulation of intracellular and mitochondrial H_2_S through the use of donor molecules or other agents which produce or are derived from H_2_S such as perthiols and persulfides may have therapeutic potential in the future for extension of health span and treatment of age-related diseases.

## MATERIALS AND METHODS

### Synthesis of H_2_S donors

**Figure 5:**
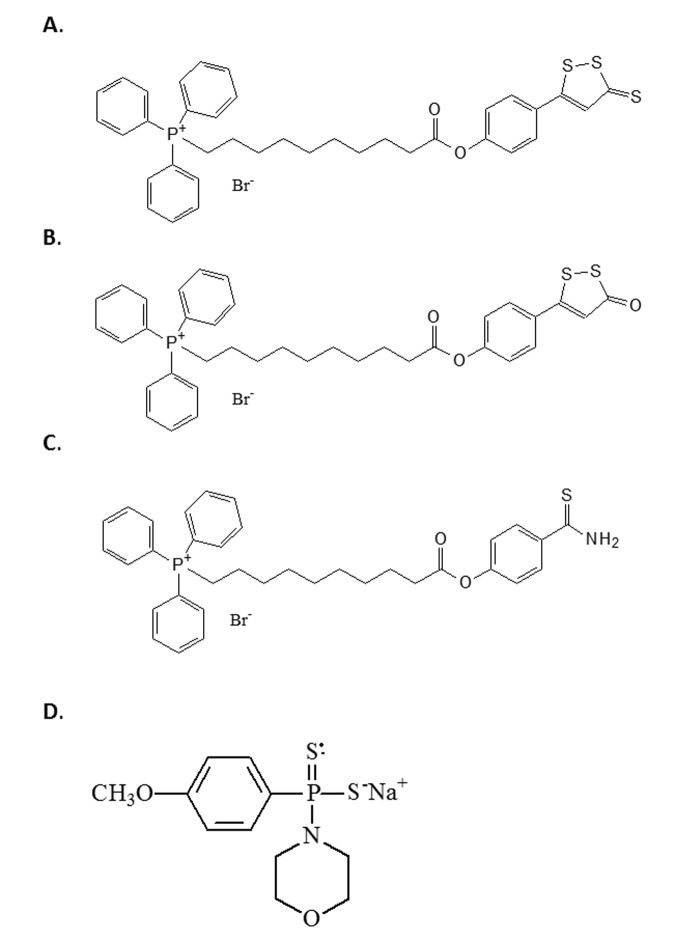
**The structure of the H2S donor compounds used in this study.** The structures of the compounds used in this study are given. (**A**) AP39 (**B**) RT01 (**C**) AP123 (**D**) Sodium GYY4137.

All H_2_S donors used in this study were synthesised in-house as previously described by us and produce physiologically-relevant levels of H_2_S [37–40]. Firstly, we used the Dichloromethane and morpholine-free GYY4137 - sodium salt, which leads to slow release H_2_S [73]. Although GYY4137 is a widely used available tool to investigate the effects of slowly generated low levels of H_2_S, it should be used with caution; commercially available GYY4137 contains an unstated amount of dichloromethane residual from initial synthesis since it is sold as a dichloromethane complex (www.sigmaaldrich.com/catalog/product/sigma/sml0100) and the molecular weight is uncertain. Dichloromethane is part of the lattice structure of the molecule at a ratio of at least 2 dichloromethane: 1 GYY4137 molecule [73] and it is difficult to remove. Indeed, to date, no published study using this compound has removed this solvent from their preparations. Furthermore, this dichloromethane is carcinogenic and metabolised in vivo to carbon monoxide, potentially producing at least 2 CO per H_2_S liberated, leading to suggestions that there may be H_2_S-independent effects of GYY4137 [74]. Furthermore, the organic counter ion (morpholine) is itself biologically active and toxic; the LD_50_ for morpholine in rodents is 300-500 mg/kg and GYY4137 is routinely used in acute studies this dose range. For the above reasons we therefore used the sodium salt of GYY4137 (Na- GYY4137) which is devoid of residual solvent and morpholine [36,73] and has superior solubility. We also used the compounds AP39 [40], AP123 [38] and RT01 [39], which are targeted to the mitochondria by virtue of conjugation of the donor molecule to triphenylpshophonium; TPP+) [40]. The half-life of H_2_S is very short [38], and the use of mitochondrial targeting allows direct delivery to the organelle of interest. The structures of the four compounds are given in Figure 5.

### Culture of human primary endothelial cells

Human Aortic Endothelial Cells (HAoEC) at PD 65 were seeded at a density of 6×10^3^ cell/cm^2^ and were cultured in endothelial cell specific growth medium (C-22022, Promocell). Cells were maintained at 37 °C and 5% CO_2_ and used when not confluent to ensure that cessation of growth was not due to contact inhibition. For the production of senescent cultures, equal numbers of cells were seeded in 3 biological replicates at each passage in continuous culture until the growth of the culture slowed to less than 0.5 PD/week; this occurred at PD=65. Cells were then treated with the H_2_S donors, AP39, AP123, RT01 (at 10 ng/ml) or Na-GYY4137 at 100 µg/ml for 24hrs, or with equal volume of vehicle.

### Assessment of cellular senescence

Cell senescence was assessed in 3 biological replicates using senescence-associated β galactosidase (SA β-Gal) assay, tested in triplicate using a commercial kit (Sigma Aldrich, UK) according to manufacturer’s instructions, with a minimum of 300 cells assessed per replicate. Senescence was also quantified in molecular terms by assessing the expression of both p14 and p16 isoforms of the *CDKN2A* gene. Total RNA (100ng) was reverse transcribed in 20 µl reactions using the Superscript III VILO kit (Thermo Fisher, Foster City USA). Expression was measured by qRTPCR relative to 3 empirically-determined endogenous control genes (*GUSB, PPIA* and *GADPH*) on the QuantStudio 12K Flex platform (Thermo Fisher, Foster City, USA). PCR reactions contained 2.5 µl TaqMan Universal Mastermix (no AMPerase) (Thermo Fisher, Foster City, USA), 900 nM of each primer, 250 nM probe and 0.5 µl cDNA in a total volume of 5 µl. PCR conditions were a single cycle of 95ºC for 10 minutes followed by 40 cycles of 95ºC for 15 seconds and 60ºC for 1 minute. Expression levels were quantified by the Comparative Ct approach, and normalised to expression levels in cells treated with carrier only.

### Determination of cell proliferation

Cell proliferation levels were assessed using Ki67 staining. HAoEC cells at PD 65 were seeded at 1 x 10^4^ cells /coverslip and after 10 days were treated with Na-GYY4137 (at 100ug/ml) or AP39, AP123, RT01 (at 10ng/ml) for 24 hours in 3 biological replicates. Cells were then fixed for 10 min with 4% v/v PFA and permeabilized with 0.025% v/v Triton and 10% v/v serum in PBS for 1 hour. Cells were incubated with a rabbit monoclonal anti-Ki67 antibody (ab16667, Abcam, UK) at 1:200 overnight at 4°C followed by FITC-conjugated secondary goat anti-rabbit (1:400) for 1 hour, and nuclei were counterstained with DAPI. Coverslips were mounted on slides in DAKO fluorescence mounting medium (S3023; Dako). The proliferation index was determined by counting the percentage of Ki67 positive cells from at least 300 nuclei from each biological replicate at 400× magnification under a Leica D4000 fluorescence microscope. Proliferation was also assessed by cell counts. For this, cells were seeded at 6 x 10^4^ cells per well into 6-well plates and cultured for 10 days then treated with each compound for 24 hours. Cell counts in three replicates of treated and vehicle-only cultures were carried out manually following trypsinisation and suspension of cells and are presented as mean (+/-SD).

### Quantification of secretion of SASP

HAoEC cells at PD 65 were seeded at 6 x 10^4^ cells per well in a 6-well plate and allowed to grow until 90% confluence. Cells were then treated with Na-GYY4137 (at 100 µg/ml) or AP39, AP123, RT01 (at 10 ng/ml) for 24 hours. Cell supernatants were harvested and stored at -80°C. Levels of 9 cytokines (GM-CSF, IFNγ, IL1β, IL2, IL6, IL8, IL10, IL-12p70, and TNFα) were measured in cell supernatants using the K15007B MesoScale Discovery multiplex ELISA immunoassay (MSD, Rockville, USA) in 6 biological replicates. Proteins were quantified relative to a standard curve using a Sector Imager SI-6000 according to the manufacturer’s instructions. Data are presented as mean (+/-SD).

### Assessment of telomere length

DNA was extracted from 2x10^5^ late passage primary human fibroblasts at PD= 63 which had been plated in 3 biological replicates and then treated with with H_2_S donors for 24hrs, using the PureLink® Genomic DNA Mini Kit (Invitrogen™/Thermo Fisher, MA, USA) according to the manufacturer’s instructions. DNA quality and concentration was checked by Nanodrop spectrophotometry (NanoDrop/Thermo Fisher, MA, USA). Relative telomere length was determined using a modified qPCR protocol [75]. PCR reactions contained 1μl EvaGreen (Solis Biodyne, Tartu, Estonia), 2μM each primer and 25ng DNA in a total volume of 5 μl in a 384 well plate. The quantitative real time polymerase chain reaction telomere assay was run on the StepOne Plus, cycling conditions were: a single cycle of 95ºC for 15 minutes followed by 45 cycles of 95ºC for 10 seconds, 60ºC for 30 seconds and 72ºC for 1 minute. The average relative telomere length was calculated as the ratio of telomere repeat copy number to a single copy number gene (*36B4*) and normalised to telomere length in untreated cells.

### Assessment of DNA damage

Cells were seeded at 1 x 104 cells /coverslip and after 10 days were treated with each compound for 24 hours in 3 biological replicates. Cells were fixed for 10 min with 4% PFA and permeabilized with 0.025% Triton and 10% serum in PBS for 1 hour. Cells were incubated with a mouse monoclonal anti-H2A.X antibody (ab26350, Abcam, UK) at 1:500 overnight at 4 °C followed by FITC-conjugated secondary goat anti-mouse (1:400) for 1 hour, and nuclei were counterstained with DAPI. Coverslips were mounted on slides in DAKO fluorescence mounting medium (S3023; Dako). The DNA damage was determined by counting the percentage of H2A.X positive cells from at least 300 nuclei from each biological replicate at 400× magnification under a Leica D4000 fluorescence microscope.

### Assessment of re-entry to S-phase

We used the BrdU cell proliferation ELISA kit (Abcam) according to the manufacturer’s instructions to assess re-entry of the cells into S-Phase. 1000 cells per well were seeded in a 96-well plate and incubated with BrdU for 24hrs. Cells were then fixed, permeabilized and the DNA was denatured by Fixing Solution according to the kit instructions. BrdU was then detected using an anti-BrdU monoclonal antibody provided in the kit, which was incubated for one hour. Unbound antibody was removed by washing, and positive cells were visualised using a horseradish peroxidase-conjugated goat anti-mouse antibody supplied with the kit. The absorbance was detected using a Pherastar plate reader at 450nm

### Assessment of ability of H_2_S donors to retard, as well as reverse senescence

Human Aortic Endothelial Cells (HAoEC) at PD 44 were seeded at a density of 6×10^3^ cell/cm^2^ and were cultured in endothelial cell specific growth medium (C-22022, Promocell). Cells were maintained at 37 °C and 5% CO_2_ and used when not confluent to ensure that cessation of growth was not due to contact inhibition. Cells were treated with H_2_S donors or vehicle only as described above in 3 biological replicates. Cells were maintained over 2 passages, then the number of senescent cells in treated and untreated cells was measured by SA-β-Gal staining as described above.

### Expression profiling of splicing factor transcripts

HAoEC cells at PD 65 were seeded at 6 x 10^4^ cells per well in 6 well plates and allowed to grow for 10 days (90% confluency) then treated with Na-GYY4137 (at 100ug/ml) or AP39, AP123, RT01 (at 10ng/ml) for 24 hours in 3 biological replicates, with vehicle only controls (DMSO). 20 splicing factors transcripts that were associated with age and replicative senescence in our previous work [18] were selected for assessment. The list of genes included the positive regulatory splicing factors *SRSF1, SRSF2, SRSF3, SRSF6, SRSF7, PNSIR* and *TRA2B*, the negative regulatory splicing inhibitors *HNRNPA0, HNPNPA1, HNPNPA2B1, HNPNPD, HNPNPH3, HNPNPK, HNPNPM, HNPNPUL2* and the core spliceosomal factors *AKAP17A, LSM2, LSM14, IMP3* and *SF3B1*. Gene expression was measured as described above, using a custom TaqMan Low Density Array (TLDA) format from Thermo Fisher (Foster City USA). Transcript expression was assessed by the Comparative Ct approach, relative to the *IDH3B, GUSB* and *PPIA* endogenous control genes and normalised to their expression in RNA from vehicle treated control cells.

### Genetic knockdown of HNRNPD and SRSF2 gene expression using morpholino oligonucleotides

We assessed the effect of knockdown of *HNRNPD* or *SRSF2* gene expression on cellular senescence and splicing factor expression in late passage primary human endothelial cells. Late passage cells at PD = 65 were seeded at 6 x 10^4^ cells per well into 6-well plates were cultured for 10 days. Antisense oligonucleotides (morpholinos, MOs) were designed to the 5’ untranslated region of the *HNRNPD* or *SRSF2* genes, in the vicinity of the initiation codon; *HNRNPD* – 5’ CCGAACTGCTCCTCCGACATAGTGC 3’ and *SRSF2* – 5’ GGCCGTAGCTCATAGCTCTGAGTGG 3’ (Gene Tools LLC, Philomath, USA). Morpholino oligonucleotides (10µM) were introduced into the cells by endo-porter delivery according to the manufacturer’s instructions. A fluorescein-conjugated scrambled negative control morpholino was also included as a negative control and to monitor delivery of constructs. Transfection efficiency (65%) and degree of knockdown was assessed by microscopy. Splicing factor expression and cellular senescence were then determined as described above.

### Statistics

Unless otherwise indicated, differences between treated and vehicle-only control cultures were assessed for statistical significance by one-way ANOVA analysis. Statistical analysis was carried out with the computer-assisted Prism GraphPad Program (Prism version 5.00, GraphPad Software, San Diego, CA).
